# Influence of Operating Temperature on the Properties and Performance of Two Heat-Treated Reciprocating NiTi Instruments: An In Vitro Study

**DOI:** 10.3390/dj14040230

**Published:** 2026-04-13

**Authors:** Tahreer Almutairi, Rashid El Abed, Anas Al-Jadaa, Amar H. Khamis, Amre R. Atmeh

**Affiliations:** 1 Hamdan Bin Mohammed College of Dental Medicine (HBMCDM), Mohammed Bin Rashid University of Medicine and Health Sciences (MBRU), Building 34, Dubai Healthcare City, Dubai P.O. Box 505055, United Arab Emiratesrashid.elabed@dubaihealth.ae (R.E.A.); amar.hassan@dubaihealth.ae (A.H.K.); 2 Department of Clinical Sciences, College of Dentistry, Ajman University, Ajman P.O. Box 346, United Arab Emirates; a.aljadaa@ajman.ac.ae; 3 Dentistry, College of Medicine and Health, University of Birmingham, Birmingham B15 2TT, UK

**Keywords:** blue heat-treated reciprocating NiTi, gold heat-treated reciprocating NiTi, cutting efficiency, intracanal temperature, bending stiffness

## Abstract

**Introduction**: Advancements in thermo-mechanical surface treatment of endodontic nickel–titanium (NiTi) instruments introduced another aspect of variation. Particularly related to their metallurgy, which influences their behaviour in relation to temperature. This is clinically significant, considering the variation in the temperatures inside the root canal during instrumentation. This study aimed to compare the effects of different temperatures on the bending stiffness, cyclic fatigue resistance, and cutting efficiency of two reciprocating heat-treated NiTi files: R-Motion (RM) and WaveOne Gold (WOG). **Methodology**: Bending stiffness was examined in a temperature-controlled water bath, measuring the maximum force in Newtons during a 3 mm tip horizontal displacement. The cyclic fatigue resistance was tested in a simulated stainless-steel canal (35° curvature, 6 mm radius) in dynamic mode at 22 °C, 37 °C, and 45 °C. Time to fracture (TTF) and length of fractured fragment were recorded, and representative samples were examined using scanning electron microscopy. The cutting efficiency was assessed using bovine bone slabs measuring 1.5 mm in thickness and 15 mm in width. The files were activated in reciprocation mode for three minutes while resting on the upper surface of the slab, while submerged in a water bath maintained at 22 °C, 37 °C, or 45 °C. The maximum cutting depth was measured in millimetres under magnification. Additionally, Differential Scanning Calorimetry (DSC) analysis was performed for three specimens of each file type. **Results**: RM exhibited significantly higher TTF, longer fractured fragments, and smaller cutting depths than WOG across all temperatures. The RM was significantly stiffer at 37 °C and 45 °C only. For each file type, increasing the temperature was associated with a significant increase in stiffness (*p* < 0.01), except for WOG between 22 °C and 37 °C (*p* = 0.199). The TTF was significantly higher in RM at 22 °C, while the TTF in WOG increased significantly with lower temperatures. No effect was observed on the length of the fractured fragment. Lower temperatures were also associated with reduced cutting efficiency in both files. **Conclusions**: Temperature has a significant impact on the properties and performance of RM and WOG and should be considered during instrumentation. File design has a greater influence on their strength and cutting ability than their transformation behaviour related to heat treatment.

## 1. Introduction

Mechanical instrumentation is an integral part of root canal treatment, which aims to prepare the canal space, reduce infected dentine, facilitate chemical disinfection, and receive a well-adapted root canal filling [1]. For this to be achieved, endodontic instruments require a combination of strength, flexibility, and efficient cutting to safely manoeuvre the narrow, curved spaces of the canals with minimal risk of overcutting and instrument separation [2]. Therefore, the introduction of nickel–titanium (NiTi) instruments has improved clinicians’ ability to achieve this, offering advantageous properties and performance. Such properties are primarily attributed to the unique crystalline structure of NiTi [3]. Hence, it can exist in a relatively rigid superelastic austenitic phase at higher temperatures and transforms into a more flexible shape memory martensitic phase when subjected to stress or when the temperature drops below the transformation range [4].

Using thermo-mechanical (heat) surface treatment, newer generations of NiTi instruments are produced with higher phase transformation temperatures [5]. This enables benefitting from the martensitic phase at higher temperatures within the clinical range, unlike conventional NiTi, which has a very low transformation temperature [6]. Heat treatment is characterised by the colour of the alloy, which typically reflects the various treatments applied and different properties [7]. Both R-Motion (RM) (FKG Dentaire SA, La Chaux-de-Fonds, Switzerland) and WaveOne Gold (WOG) (Dentsply Sirona, Ballaigues, Switzerland) are reciprocating single-file systems, but with different heat treatments. The blue heat-treated RM exhibits its colour from the titanium oxide layer formed on the surface after the machining process [8]. At the same time, the gold heat-treated WOG is produced by heating M-wire NiTi, followed by a slow cooling procedure [9]. The constant taper RM files exhibit an austenite finish (A_f_) temperature lower than that of the variable taper WOG [10,11]. Compared to gold heat-treated and conventional NiTi files, the blue heat-treated file exhibited superior cyclic fatigue resistance [11,12,13].

The relation between temperature and the crystallographic status of NiTi highlights the importance of operating temperature on the file during instrumentation inside the canal [14]. This is particularly important as the operating temperature in the root canal is not consistent and varies depending on the temperature of the irrigation solution [15]. Depending on the temperature of the irrigation solution, files may be subjected to temperatures ranging from room temperature to 45 °C or higher if a heated solution is used [16]. Therefore, unlike what is assumed, the intracanal temperature, which represents the operating temperature, is not fixed at the body temperature, as the time needed for the temperature to equalise with body temperature is typically longer than the time the instrumentation takes place [15].

Having different types of NiTi instruments with varying transformation temperatures means that their operating temperature can influence their properties and performance differently [4,17,18,19]. With higher temperatures, more austenite can be present in the NiTi alloy, resulting in a stiffer instrument that is more prone to separation due to reduced fatigue resistance [20]. However, a comprehensive understanding of the effects of various temperatures within the clinically used range on the properties and performance of different heat-treated NiTi instruments is lacking. Particularly in relation to the effect of temperature on the performance and cutting efficiency of these instruments, which emphasises the need for further investigation in this area [21].

This study aimed to compare the effects of different operating temperatures on the bending stiffness, cyclic fatigue resistance, and lateral cutting efficiency of two types of reciprocating NiTi instruments: blue heat-treated RM and gold heat-treated WOG. The null hypotheses were: (1) operating temperatures of 22 °C, 37 °C, and 45 °C have no significant effect on the cyclic fatigue resistance, bending stiffness, or lateral cutting efficiency of each tested reciprocating file (RM and WOG); and (2) there are no significant differences between the two files at each tested temperature.

## 2. Materials and Methods

Two types of reciprocating single NiTi file systems: blue heat-treated R-Motion (RM) file size 25/0.06 (FKG, La Chaux de Fonds, Switzerland) and gold heat-treated WaveOne Gold (WOG) primary file (Dentsply Sirona, Ballaigues, Switzerland) were tested at three different temperatures: 22 °C, 37 °C, 45 °C. A new file of each type was imaged under magnification using a stereomicroscope (Optika SZX-T, Ponteranica, Italy) equipped with a digital camera (Optika C-HP4, Ponteranica, Italy). The files were then sectioned horizontally at 3 mm, 6 mm, 9 mm, and 16 mm from the tip before imaging.

### 2.1. Differential Scanning Calorimetry (DSC)

Three instruments from each file type were tested. Fragments of 2–4 mm length weighing 5–10 mg were prepared. Samples were separately encased in aluminium pans and loaded into a DCS device (DSC 25, TA Instruments, New Castle, DE, USA). Samples were heated initially from room temperature to 150 °C, cooled down to 70 °C, and heated again to 150 °C at a rate of 10 °C/min. Cooling was performed using a refrigerated cooling system with a nitrogen purge of 50 mL/min. Thermogram plots were created with TRIOS v5.0.0.44608 software. The phase transformation temperatures—martensite start (M_s_), martensite finish (M_f_), austenite start (A_s_), and austenite finish (A_f_)—were identified at the intersection points of the steepest slope and the baseline on the DSC curve.

### 2.2. Bending Stiffness Test

**Figure 1:**
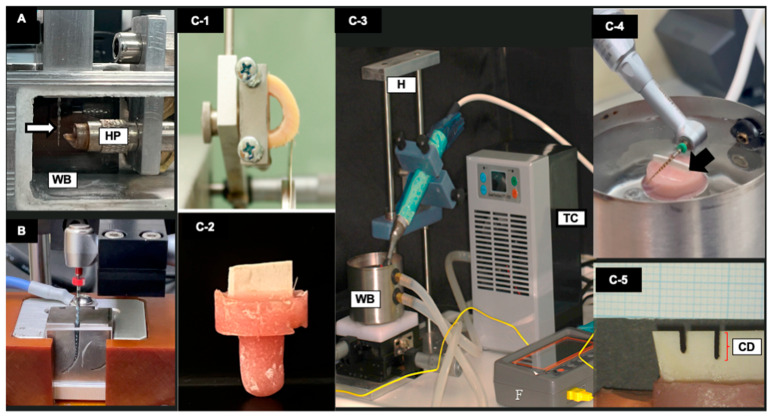
Experimental set-up: (**A**) Bending stiffness test equipped with a temperature-controlled water bath (WB) with a horizontal plunger (HP) in contact with the tested file (white arrow). (**B**) Cyclic fatigue test using EndoC equipped with a simulated canal of tempered steel with temperature control. (**C**) Lateral cutting efficiency. The shaft of a bovine femur bone was horizontally cut into 2 cm thick sections (**C-1**). Bone slabs of 1.5 mm thickness and 15 mm width were cut and mounted on acrylic stumps (**C-2**). Each file was attached to a free-falling handpiece, perpendicular to the sample, 15 mm from the tip, allowing for free vertical movement (**C-3**). The samples were fixed in a temperature-controlled water bath (WB) while the file was in direct contact with the upper surface of the bone slab, submerged in water during activation (**C-3**,**C-4**). The cutting depth (CD) was measured under 8× magnification from the upper surface of the bone slab to the deepest cutting point (**C-5**).

A custom-made bending stiffness testing device was used, equipped with a temperature-controlled water bath and a constant temperature monitor via a K-type thermocouple thermometer (THE-315_2P Thermocouple Temperature K Type Thermometer, Hong Kong, China) (Figure 1A). Six samples (*n* = 6) were tested from each file type at each temperature: 22 ± 1 °C, 37 ± 1 °C, and 45 ± 1 °C. Each sample was mounted into a vertical fixture while a horizontal stainless-steel plunger was placed in contact with the file 3 mm from its tip. The horizontal plunger was attached to a force gauge (Imada DS2, Imada Inc., Northbrook, IL, USA), enabling real-time measurement of force changes during displacement. The plunger was activated for 30 s, which was needed to displace the tip by 3 mm, and the maximum force was recorded in Newtons (N).

### 2.3. Cyclic Fatigue Testing

For each file, the cyclic fatigue resistance was evaluated in a simulated canal of tempered steel with a 17 mm length and a 6 mm curvature radius (35° angle) in a dynamic mode using the EndoC testing device (DMJ System, Busan, Republic of Korea) (Figure 1B). A synthetic lubricant (WD-40, San Diego, CA, USA) was applied to reduce friction between the file and canal walls. Files were activated using an endodontic motor (X-Smart Plus, Dentsply Sirona, Ballaigues, Switzerland) in a reciprocating motion (150° CCW and 30° CW). Dynamic testing involved a 4 mm pecking motion every 0.5 s, with a 50 ms dwell time. Tests were performed at three temperature settings: 22 °C (room temperature, maintained in an air-conditioned room), 37 °C, and 45 °C (using a heat control unit (TK4N/S/SP; Autonics, Busan, Republic of Korea). The temperature was constantly monitored using a thermal FLIR camera (FLIR Systems OU, Tallinn, Estonia). Temperature was continuously monitored throughout testing using a K-type thermocouple and verified in real time with FLIR thermal imaging to maintain it within ±1 °C of the target value. The time to fracture (TTF) was recorded for each file using a chronometer and confirmed by video capture. The length of fractured fragments was measured using a digital microcaliper (Mitutoyo, Kanagawa, Japan).

### 2.4. Scanning Electron Microscopy (SEM)

Selected representative fractured files from the cyclic fatigue test were examined using SEM (COXEM Co. Ltd., Daejeon, Republic of Korea) operated at an accelerating voltage of 15 kV and a working distance of 10–15 mm. Images were obtained at magnifications ranging from ×100 to ×500 to examine the topographic characteristics of the instruments’ fractured surfaces, including crack initiation areas, fatigue striations, and final overload zones with ductile dimples. This was used to e. The fractured files were carefully mounted on aluminium stubs using double-sided carbon-based conductive adhesive film. Images were obtained for the fractured and longitudinal surfaces of the fractured files.

### 2.5. Cutting Efficiency

Femur bovine bone obtained from animals that were slaughtered for food production by a certified provider, which was approved by the local ethics committee. The shaft of the femur bone was horizontally cut into 2 cm sections. All attached ligaments, bone marrow, and soft tissues were removed. Each section was cut vertically into two halves using a water-cooled wafering blade (IsoMet, Buehler, Lake Bluff, IL, USA). Bone slabs of 1.5 mm thickness and 15 × 10 mm (width × height) were cut and mounted on acrylic stubs perpendicular to the base, ensuring that the flat sectioned surface of the bone was aligned parallel to the base (Figure 1C).

Cutting efficiency was evaluated following a previously published methodology [22]. Each file was attached to a free-falling handpiece with a 2.7 N load, positioned perpendicularly to a bovine bone slab, which was submerged in a temperature-controlled water bath. The files were positioned 15 mm away from the tip and allowed free vertical movement during activation. Files were operated in reciprocating motion (150° CCW and 30° CW) using the X-Smart Plus endodontic motor for 3 min.

Water temperature was regulated and circulated using a control unit (GeekTeches, Xuzhou Sanhe Automatic Control Equipment Co., Ltd., Xuzhou, China) and monitored using a K-type thermocouple thermometer (THE-315_2P, Hong Kong, China). Digital images were captured of the cut samples at 8× magnification using an operating dental microscope (Leica M320, Wetzlar, Germany), with 1 mm graph paper placed beside it for calibration. Using the calibrated measuring tool of image analysis software (ImageJ, v1.53t, NIH, Bethesda, MD, USA), the cutting depth of each file was measured as the straight distance from the upper surface of the slab to the deepest point of penetration. Measurements were performed by two evaluators and repeated three times, one week apart.

### 2.6. Statistical Analysis

The Statistical Package for the Social Sciences (SPSS) v25.0 (SPSS Inc., Chicago, IL, USA) was used for data entry and statistical analysis. The normality of data distribution was evaluated using the Shapiro–Wilk test. Two-way analysis of variance (ANOVA) was used to compare the effect of temperature and file type on the bending stiffness, cyclic fatigue resistance, fractured fragment length, and cutting depth. Based on the normality of the data distribution, parametric (one-way ANOVA and *t*-test) or non-parametric (Kruskal–Wallis and Mann–Whitney) tests were used.

## 3. Results

**Figure 2:**
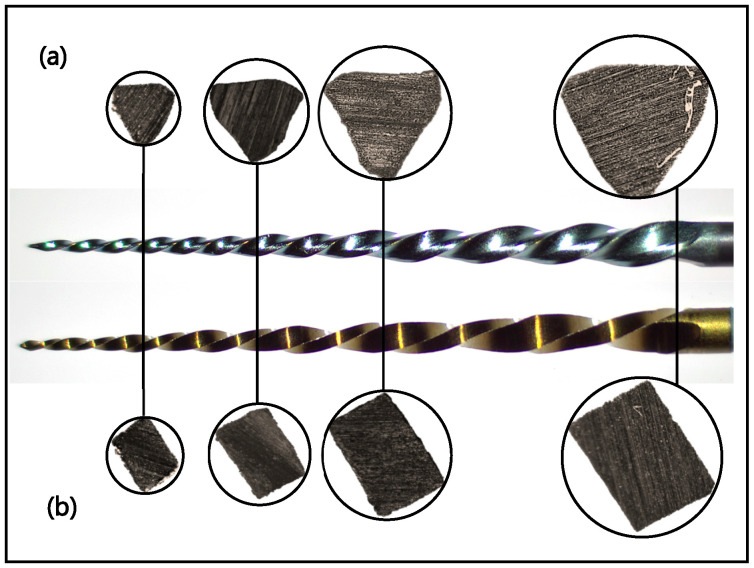
Geometric design of blue heat-treated RM (**a**), and gold heat-treated WOG (**b**), demonstrating the longitudinal and cross-section shape at 3, 6, 9, and 16 mm.

The longitudinal and cross-sectional appearances at 3 mm, 6 mm, 9 mm, and 16 mm of both file types are shown in Figure 2, demonstrating the differences in geometric design between the two files.

### 3.1. Differential Scanning Calorimetry

**Figure 3:**
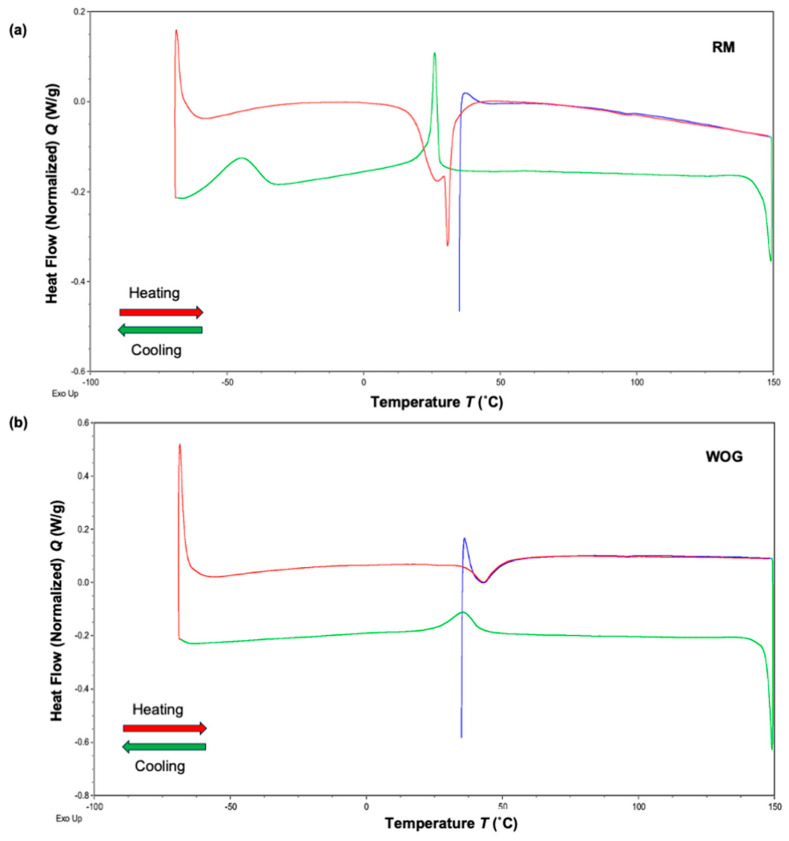
DSC thermograms of RM (**a**) and WOG (**b**) showing the martensitic and austenitic phase transformations. The exothermic forward transformation upon cooling (green) and the en-dothermic reverse transformation upon heating (blue, red) are shown. The characteristic transformation start and finish temperatures (M_s_, M_f_, A_s_, A_f_) are determined by the tangent method. Exothermic heat flow is upward.

The DSC thermograms displaying the heating and cooling cycles for both files are shown in Figure 3. The results confirm a reversible thermoelastic martensitic transformation, indicated by the clear exothermic peaks during cooling and endothermic peaks during heating. The phase transformation temperatures are listed in Table 1.

In the heating curves, two prominent endothermic peaks were noted in RM, while WOG showed only one. The A_f_ temperatures for RM and WOG were 33 ± 0.8 °C and 49.2 ± 0.5 °C, respectively. During cooling, RM exhibited two exothermic peaks, whereas WOG showed a single peak, suggesting a phase change from austenite to martensite. The M_f_ temperatures measured for RM and WOG were 23.9 ± 0.5 °C and 31.0 ± 0.2 °C, respectively.

### 3.2. Bending Stiffness

The maximum force recorded during tip displacement for each file type at different temperatures is shown in Table 1. The two-way ANOVA revealed significant effects of file type (*p* < 0.001) and operating temperature (*p* < 0.001). Lower temperatures were associated with significantly lower stiffness. The RM file exhibited greater stiffness than WOG, which was statistically significant at higher temperatures of 37 °C (*p* = 0.002) and 45 °C (*p* = 0.002), but not at 22 °C (*p* = 0.199). For the blue heat-treated RM, stiffness was temperature-dependent, with the lowest value at 22 °C, followed by 37 °C and 45 °C, respectively (*p* < 0.001). For the gold heat-treated WOG, the file’s stiffness was also affected by temperature, showing significant differences between all temperatures except between 22 °C and 37 °C (*p* = 0.385).

### 3.3. Cyclic Fatigue

**Figure 4:**
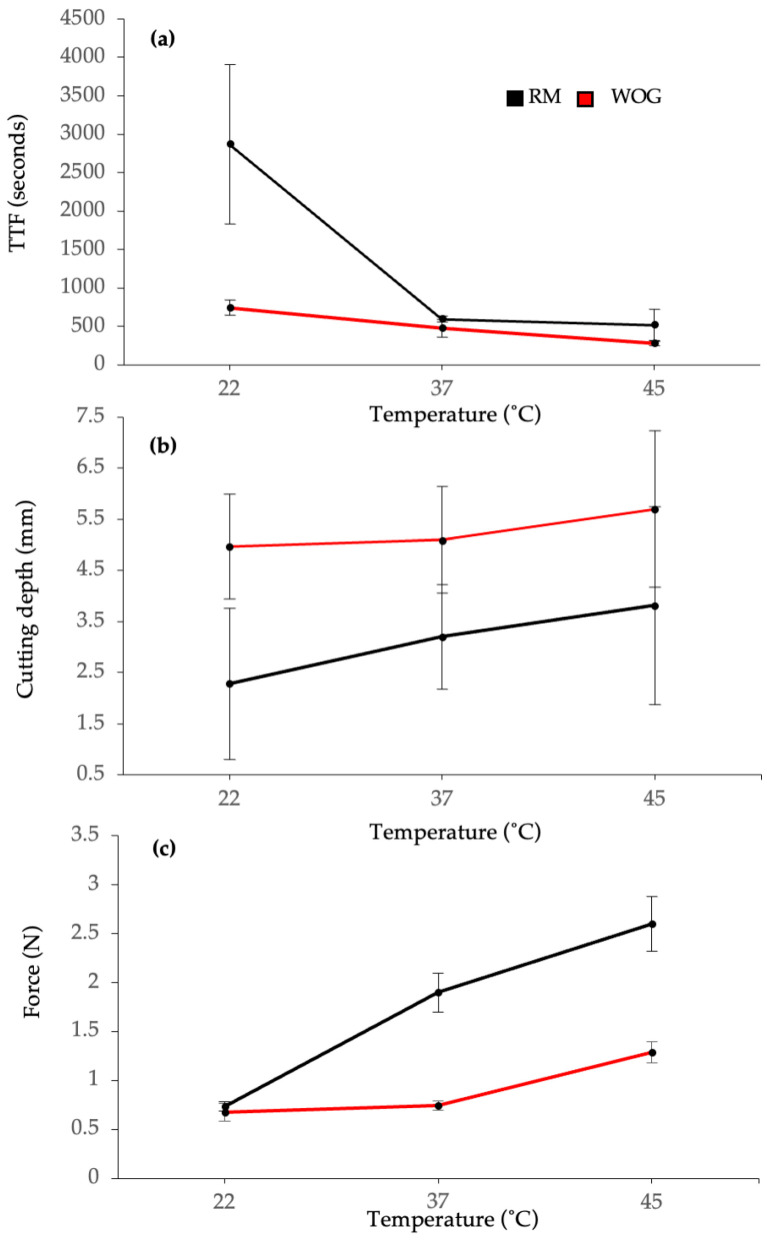
The effect of operating temperature on the cyclic fatigue resistance (**a**), cutting efficiency (**b**), and bending stiffness (**c**) of blue (RM) and gold (WOG) heat-treated NiTi files.

The TTF and fractured fragment lengths for both RM and WOG files at different temperatures are presented in Table 1 and Figure 4. According to two-way ANOVA, file type and temperature significantly affected cyclic fatigue resistance (*p* < 0.001); the interaction between these two variables was also significant (*p* < 0.001). Comparing the mean TTF values between the two file types at each temperature revealed that RM had significantly greater cyclic fatigue resistance than WOG at all temperature levels (*p* < 0.005). Lower temperatures were associated with significantly higher TTF and fracture resistance (*p* < 0.001). For RM, the TTF was significant across all temperatures except between 37 °C and 45 °C (*p* = 958), whereas for WOG, the TTF varied significantly across all temperatures (*p* < 0.001).

For the fractured fragment length, only the file type was found to have a significant effect, with the RM exhibiting longer fractured fragments (*p* < 0.001). In contrast, no significant effect of the operating temperature was observed (*p* = 0.306). Using Mann–Whitney’s test for pairwise comparisons, the fragment length of RM was significantly higher than WOG at all temperature levels: 22 °C (*p* = 0.001), 37 °C (*p* = 0.029), and 45 °C (*p* < 0.001).

**Figure 5:**
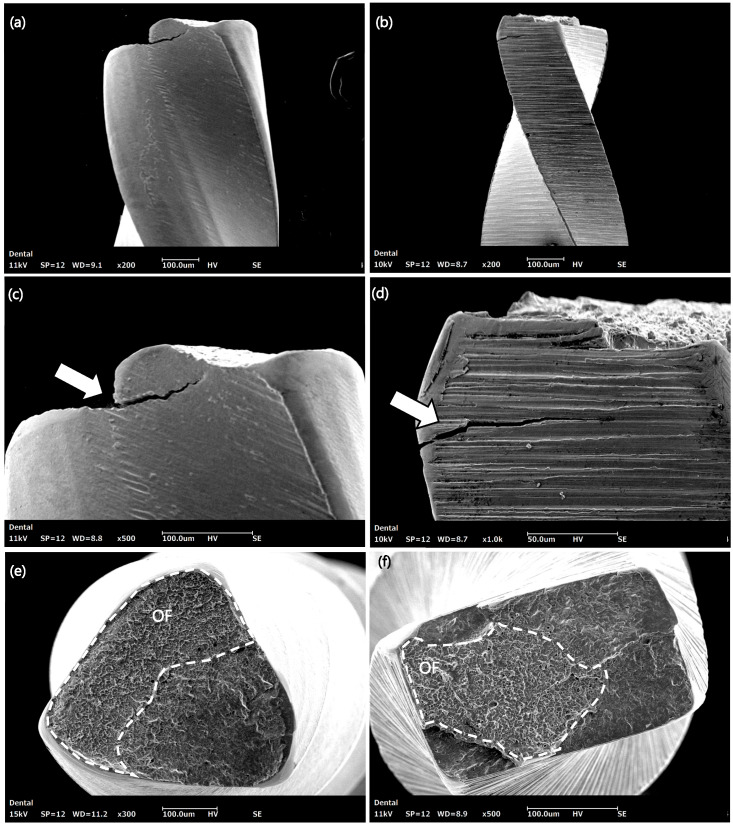
Scanning electron micrographs of the fracture surfaces after cyclic fatigue resistance tests showing longitudinal (**a**–**d**) and cross-sectional (**e**,**f**) views of the fractured instruments: RM (**a**,**c**,**e**) and WOG (**b**,**d**,**f**). Microcracks are detectable at higher magnification (**c**,**d**) near the fracture point (white arrows). The RM instruments (**a**,**c**) exhibit a smoother surface than the WOG (**b**,**d**), with deep milling grooves that correspond to the initiation and propagation of microcracks. The outlined area in (**e**,**f**) indicates the overload fast fracture with ductile dimples.

A fractographic analysis of the samples that underwent the cyclic fatigue testing revealed characteristic features confirming cyclic fatigue failure. This includes the presence of fatigue striations along the peripheries and a broad, fibrous region situated at the core of the specimens, visible at high magnification levels (Figure 5).

### 3.4. Cutting Depth

Using repeated measurement ANOVA, the readings were considered reliable since the inter- and intra-examiner reliability was not found to be statistically significant (*p* = 0.791). The mean values of the cutting depth associated with RM and WOG at different temperatures are shown in Table 1 and Figure 4. Based on the two-way ANOVA, significant effects of file type (*p* < 0.001) and operating temperature (*p* = 0.042) were found on cutting depth, with no significant interaction between the two independent variables (*p* = 0.576). The results of the t-independent analysis, used to compare the two instruments, revealed that the cutting depth differed significantly between RM and WOG at each temperature (*p* < 0.001), with RM exhibiting a lower cutting depth. Although the temperature was found to affect the cutting depth of files, when the cutting depth was assessed within each file group at different temperatures using one-way ANOVA, no significant differences were found.

## 4. Discussion

This study compared the effect of different temperatures on the bending stiffness, cyclic fatigue resistance, and cutting efficiency of blue heat-treated (RM) and gold heat-treated (WOG) reciprocating files. Based on our findings, there was a significant effect of the temperature on these factors, which rejects the first null hypothesis. The highest temperature was associated with increased stiffness, reduced cyclic fatigue resistance and lateral cutting efficiency, while no effect was observed on the length of the fractured fragment. Furthermore, the file type was also found to have a significant effect on these three factors, which rejects the second null hypothesis. The RM was associated with greater stiffness, higher cyclic fatigue resistance, longer fractured fragments, and a lower cutting efficiency compared to the WOG.

In both files, the temperature significantly affected stiffness, which is expected according to their DSC analysis and transformation behaviour. At 22 °C, both files were entirely in a martensitic phase; thus, they exhibited similar flexibility. At 37 °C, the WOG remained martensitic (M_S_ = 41.2 °C), unlike the RM (A_f_ = 33 °C); consequently, the latter was stiffer. At 45 °C, the WOG showed lower stiffness than the RM, as it contained a lower proportion of austenitic phase. Interestingly, the RM had significantly higher stiffness at 45 °C compared to 37 °C, even though the blue heat-treated file was expected to be fully austenitic at both temperatures. This may be due to the proximity of the 37 °C temperature to the A_f_, or more likely, due to the effect of temperature on stress-induced transformation, which requires higher stress to induce martensitic transformation at higher temperatures [5]. A study by Hou et al. reported no significant difference between WOG and blue heat-treated Reciproc Blue [23]. This agrees with our findings, as their test was conducted at room temperature. However, the difference in stiffness between the two files was particularly noticeable at higher temperatures. It highlights the importance of considering different temperatures during in vitro assessment of these files [15].

The blue heat-treated RM file demonstrated superior cyclic fatigue resistance compared to WOG, regardless of temperature, which aligns with a recent study that compared the same files [13]. This can partly be attributed to the file’s design [23]. The RM features a triangular cross-section with a smaller core diameter than the parallelogram cross-section of the WOG (Figure 2). Another factor could be the different thermo-mechanical surface treatments [11,13]. The presence of two different file colours suggests different thermo-mechanical treatments and, therefore, different phase transformation behaviours and properties [23,24]. However, this is questioned given the higher stiffness of the RM at increased temperatures, which is expected to have an inverse effect on cyclic fatigue resistance (Figure 4). Furthermore, the RM exhibited significantly higher cyclic fatigue resistance than WOG at all tested temperatures, emphasising that file design plays a more crucial role than metallurgy and transformation behaviours in determining cyclic fatigue resistance [23]. Surface electropolishing might also contribute to the enhanced cyclic fatigue resistance of RM [13]. Having fewer surface defects provides the instrument with improved resistance to fracture, as shown in the SEM micrographs (Figure 5)

Temperature significantly affected the cyclic fatigue resistance of RM files, with TTF dropping about 80% when the temperature increased from 22 °C to 37 °C or 45 °C. The DSC analysis confirmed the blue heat-treated file had an A_f_ temperature of 33 °C, indicating that the file had fully transformed into the austenitic phase, exhibiting nearly the same behaviour at 37 °C and 45 °C [11]. Whereas at 22 °C, the file was mainly in the martensitic phase (M_f_ = 23.9 °C), which explains its higher cyclic fatigue resistance due to the increased elasticity of NiTi at this phase. The presence of two endothermic and exothermic peaks in RM thermograms suggests an intermediate R-phase transformation prior to the martensite–austenite transformation, which has been described in thermo-mechanically treated NiTi alloys [5,24]. In the WOG, the TFF dropped by 32% and 65% when the temperature increased from 22 °C to 37 °C and 45 °C, respectively (Figure 4). According to the DSC analysis, the gold heat-treated file was in a complete martensitic phase at 22 °C (M_f_ = 31 °C). At temperatures of 37 °C and 45 °C, the file was exposed to conditions below the Af (49.2 °C), suggesting a mixture of austenite and martensite phases at different temperature levels, with a higher proportion of austenite as the temperature increases, resulting in a gradual decrease in TTF as the temperature increases (Figure 4) [25]. Similar results showing the effect of temperature on cyclic fatigue resistance have been reported in previous studies [4,17,18,19].

Regardless of the temperature, RM had significantly longer fractured fragment length, which agrees with a previous study [13]. This can also be attributed to the same factors discussed above, mainly in relation to file geometry and surface electropolishing.

Results from the cutting efficiency test showed significantly greater cutting depth with the gold heat-treated, regardless of the temperature (Table 1). Our findings are comparable to a recent study by Alsuleiman et al. [26], who reported a significantly greater reduction in dentine thickness with WOG compared to RM, as measured using micro-computed tomography (micro-CT). Such differences can be attributed to the geometric designs of these files. In their study, Carvalho et al. found that RM caused less dentine and canal volume reduction than Reciproc Blue, despite both being blue heat-treated [27]. They attributed this to the lower metal volume, smaller taper, and different cross-section of RM. On the contrary, comparing the cutting efficiency of two additional heat-treated blue files (E3 Zure and Fanta AF) revealed significant differences [28]. Furthermore, no significant differences were found in cutting efficiency when comparing heat-treated Reciproc Blue with non-heat-treated Reciproc files [29]. This suggests that the various file designs may have contributed to the superior cutting efficiency of the WOG, which features a higher taper and metal mass volume. Additionally, WOG’s parallelogram shape cross-section with two cutting edges may enhance cutting and debris removal. This can be further supported by comparing the cutting efficiency of the same file at different temperatures, which showed no significant differences despite the phase transformations. However, this cannot rule out the possible effect of heat treatment on enhancing cutting efficiency, which is explained by the increased contact area with the substrates, promoting more efficient cutting than conventional NiTi [30,31]. Although this might be questioned, considering that both files had similar stiffness at 22 °C (Figure 4). Hence, WOG’s greater cutting depth supports that design, rather than metallurgy and transformation behaviour, influences the file’s cutting ability.

To the best of our knowledge, the effect of operating temperature on the cutting efficiency has not been studied. Our results showed that temperature had a significant effect on lateral cutting efficiency, as determined by the two-way ANOVA, with the highest temperature associated with the greatest cutting depth, suggesting a tendency for improved lateral cutting efficiency (Figure 4). However, when assessed within each file, one-way ANOVA found no significant effect. This discrepancy likely involves an interaction between temperature and file type, but since the interaction was insignificant, it cannot explain the results. The two-way model has more power because it accounts for variance explained by file Type, reducing the “error” term for testing the temperature effect. This boosts the chance of detecting a real effect that less powerful one-way tests on smaller datasets might miss.

Based on our findings, the highest temperature was associated with the most significant cutting depth in both files. This can be explained by the effect of temperature on the crystallographic status of NiTi in both files [32]. Based on the DCS analysis, the blue- and gold heat-treated files were fully or mostly transformed into the austenitic phase at 45 °C, respectively (Figure 3). This explains the reduction in the ratio of cutting depth between the two files, as both files adopt similar metallurgy at 45 °C, which was also confirmed by the increased stiffness of both file types at this temperature (Figure 4). Therefore, despite the primary role of geometry and design in influencing the cutting efficiency of heat-treated files, temperature can still affect their cutting ability, particularly at lower temperatures, where more martensitic phase exists, resulting in lower hardness and higher flexibility [33]. This challenges the assumption that heat treatment improves cutting efficiency, which warrants further investigation [31].

Although this study offered valuable insights into the impact of temperature on the cyclic fatigue resistance and cutting efficiency of heat-treated NiTi files, it is essential to recognise limitations, one of which is its in vitro nature. The limitations inherent to in vitro testing and their significance have been thoroughly discussed, addressing the disparity between the performance and behaviour of instruments in laboratory settings versus the oral cavity [34,35]. Consequently, this investigation was conducted considering clinically relevant temperatures, reflecting the temperature range within the root canal during procedures [15,36,37]. It should be noted, however, that these temperatures do not account for other factors that may influence them. Factors such as frictional heat generated during cyclic fatigue testing, the geometric design of the instruments, and the degree of canal curvature were all predicted to affect temperature, resulting in temperature gradients along the instrument [38]. Another limitation involved using bovine bone as a substrate to assess cutting efficiency, rather than human dentine. Although human dentine would have more closely approximated the clinical situation, bovine bone was deemed appropriate due to its comparable hardness and mechanical properties, its availability, and, most importantly, its capacity for standardisation and comparability across groups [39], a method previously utilised in various studies [39,40]. Bovine cortical bone exhibits hardness and elastic modulus values within the ranges reported for human dentin and has been validated as a standardised substrate for assessing cutting efficiency [39,40]. Furthermore, the study’s lateral cutting efficiency test may not fully reflect clinical performance, as it does not account for axial cutting, which could offer a more accurate evaluation of instrument behaviour in root canals.

In this study, a multimethod approach was employed to examine the thermal behaviours, mechanical properties, and performance of the tested files. This can be considered one of the study’s strengths. More importantly, all these tests were performed under controlled temperatures that covered the clinical range, which has been considered before. The 45 °C and 22 °C temperatures represented intracanal temperatures that simulated clinical conditions when irrigation solutions were used with or without heating, respectively. Although equalisation with body temperature will eventually occur, the time required for this to happen is much longer than the duration for which the instruments are activated within the canals [15]. This makes the irrigation solution the main determinant of the intracanal temperature during instrumentation [15]. The utilisation of DSC analysis, in conjunction with bending stiffness, cyclic fatigue resistance, and cutting efficiency testing, has provided a better and more comprehensive understanding with a broader insight into the correlation between the metallurgical and thermal transformation behaviour of tested NiTi files detected through the DSC with their mechanical properties and performance according to temperature [38].

The significant influence of irrigation solution temperature, and hence intracanal temperature, on the mechanical behaviour of nickel–titanium (NiTi) rotary instruments necessitates a strategic approach to clinical irrigation protocols. Our findings demonstrate that while the high temperature did not significantly alter the files’ cutting efficiency, it significantly reduced cyclic fatigue resistance by promoting the transition of the alloy from a flexible martensitic phase to a more rigid austenitic phase, which may also affect their shaping ability and the preservation of the original canal shape. Consequently, to optimise the benefits of heat-treated files, clinicians should consider delaying the use of heated irrigation solutions until the final stages of canal preparation. By keeping a lower intracanal temperature during active shaping, the clinician can take advantage of the increased flexibility and fatigue resistance of heat-treated NiTi files, ensuring greater safety without sacrificing the efficiency of dentin removal.

## 5. Conclusions

Within the limitations of this study, the operating temperature can exert a notable influence on the cyclic fatigue resistance and cutting efficiency of both blue- and gold heat-treated NiTi reciprocating files. However, despite the evident effect of temperature, the file design and geometry seem to have a more critical role in influencing the performance and behaviour of these files. Clinically, clinicians should consider temperature as a factor when using these instruments, as at high temperatures, these files may behave as austenitic superelastic files without benefiting from the advantages of heat treatment. While heated irrigation solutions may offer a safer alternative to higher concentrations of NaOCl, it is crucial to acknowledge the potential impact on the cyclic fatigue resistance of heat-treated endodontic files. Further research is needed to investigate the impact of heat treatment on the properties and performance of NiTi files at various temperatures.

## Figures and Tables

**Table 1:** The mean (±STD) results for cyclic fatigue resistance, cutting efficiency, and bending stiffness tests, along with transformation temperatures from DSC.

Operating Temperature	Cyclic Fatigue Resistance				Cutting Efficiency		Stiffness		DSC					
	TTF (sec)		Fragment Length (mm)		Depth (mm)		Bending Force (N)		Heating Curve			Cooling Curve		
	RM	WOG	RM	WOG	RM	WOG	RM	WOG	°C	RM	WOG	°C	RM	WOG
**22 °C**	2865.2 ^a*^ (1036.9)	743.9 ^a^ (101.3)	3.90 ^a*^ (1.06)	2.29 ^a^ (0.51)	2.28 ^a*^ (1.48)	4.97 ^a^ (1.02)	0.74 ^a^ (0.05)	0.68 ^a^ (0.09)	A_S_	30.2 (0.6)	39.0 (0.3)	M_S_	28.0 (0.6)	41.2 (0.5)
**37 °C**	597.0 ^b*^ (39.0)	481.7 ^b^ (115.2)	3.63 ^a*^ (0.98)	2.76 ^a^ (0.12)	3.20 ^a*^ (1.02)	5.09 ^a^ (1.04)	1.90 ^b*^ (0.2)	0.75 ^a^ (0.05)	A_P_	31.2 (0.6)	43.1 (0.1)	M_P_	26.5 (0.5)	35.6 (0.2)
**45 °C**	517.0 ^b*^ (210.0)	282.4 ^c^ (34.5)	3.60 ^a*^ (0.75)	2.22 ^a^ (0.88)	3.81 ^a*^ (1.93)	5.70 ^a^ (1.53)	2.60 ^c*^ (0.28)	1.29 ^b^ (0.11)	A_F_	33 (0.8)	49.2 (0.5)	M_F_	23.9 (0.5)	31.0 (0.2)

Different lowercase superscript letters in the same column indicate statistical significance at *p* ≤ 0.05. (*) Indicate statistically significant difference comparing the mean values of both file types in the same raw for the same test (*p* ≤ 0.05).

## Data Availability

The raw data supporting the conclusions of this article will be made available by the authors on request.

## Abbreviations

The following abbreviations are used in this manuscript:

DSC	Differential Scanning Calorimetry
SEM	Scanning Electron Microscopy
RM	R-Motion file
WOG	WaveOne Gold file
NiTi	Nickel–Titanium
A_f_	Austenite finish temperature
A_s_	Austenite start temperature
M_f_	Martensite finish temperature
M_s_	Martensite start temperature
